# Zip4/Spo22 Is Required for Class I CO Formation but Not for Synapsis Completion in Arabidopsis thaliana

**DOI:** 10.1371/journal.pgen.0030083

**Published:** 2007-05-25

**Authors:** Liudmila Chelysheva, Ghislaine Gendrot, Daniel Vezon, Marie-Pascale Doutriaux, Raphaël Mercier, Mathilde Grelon

**Affiliations:** 1 Institut Jean-Pierre Bourgin, INRA de Versailles, Station de Génétique et d'Amélioration des Plantes UR-254, France; 2 Université Paris XI, IBP, CNRS UMR8618, Orsay, France; Stowers Institute for Medical Research, United States of America

## Abstract

In budding yeast meiosis, the formation of class I interference-sensitive crossovers requires the ZMM proteins. These ZMM proteins are essential in forming a mature synaptonemal complex, and a subset of these (Zip2, Zip3, and Zip4) has been proposed to compose the core of synapsis initiation complexes (SICs). Zip4/Spo22 functions with Zip2 to promote polymerization of Zip1 along chromosomes, making it a crucial SIC component. In higher eukaryotes, synapsis and recombination have often been correlated, but it is totally unknown how these two processes are linked. In this study, we present the characterization of a higher eukaryote SIC component homologue: *Arabidopsis* AtZIP4. We show that mutations in *AtZIP4* belong to the same epistasis group as *Atmsh4* and eliminate approximately 85% of crossovers (COs). Furthermore, genetic analyses on two adjacent intervals of Chromosome I established that the remaining COs in *Atzip4* do not show interference. Lastly, immunolocalization studies showed that polymerization of the central element of the synaptonemal complex is not affected in *Atzip4* background, even if it may proceed from fewer sites compared to wild type. These results reveal that Zip4 function in class I CO formation is conserved from budding yeast to *Arabidopsis.* On the other hand, and contrary to the situation in yeast, mutation in *AtZIP4* does not prevent synapsis, showing that both aspects of the Zip4 function (i.e., class I CO maturation and synapsis) can be uncoupled.

## Author Summary

During meiosis two successive chromosomal divisions follow a single S phase, resulting in the formation of four haploid cells, each with half of the parental genetic material. This ploidy reduction occurs during the first meiotic division, when homologous chromosomes (paternal and maternal) are separated from each other. For this to happen, homologous chromosomes associate in bivalents, where each chromosome is linked to its homologue by chiasmata. These chiasmata reflect the formation of crossovers, one of the manifestations of the exchange of genetic material occurring during homologous recombination. Another important feature of the meiotic prophase is the transitory setup (synapsis), between homologous chromosomes, of a tripartite protein structure called the synaptonemal complex, amazingly conserved among species, but which function remains a puzzle despite half of a century of extensive survey. In this study, we investigate the relationships between these two crucial meiotic events using the model plant *Arabidopsis thaliana.* We show that in this plant, crossover formation and synapsis completion can largely be uncoupled.

## Introduction

During meiosis two successive chromosomal divisions follow a single S phase, allowing the transition from the sporophytic to the gametophytic state. This ploidy reduction occurs during the first meiotic division, when homologous chromosomes are separated from each other. For this to happen, homologous chromosomes must first associate in bivalents, linked by chiasmata—the cytological reflection of crossovers—that are established during meiotic prophase. Meiotic recombination is initiated by the induction of DNA double-strand breaks (DSBs) subsequently resected to generate 3′ single-stranded tails that invade the intact DNA duplexes that are used for DNA repair. Most of these events happen using the homologous chromosome as the template for DNA repair, to yield either crossover (CO) or noncrossover recombinant products [1].

In most organisms, the occurrence of a CO inhibits the occurrence of another event in a distance-dependent manner, resulting in COs more evenly spaced than would be expected if they occurred randomly. This phenomenon is known as interference [2]. At least two kinds of COs can coexist. In *Saccharomyces cerevisiae,* class I COs are interference-sensitive and their formation is dependent on the ZMM proteins (Zip1, Zip2, Zip3, Zip4, Msh4, 5, and Mer3) [3]. Class II COs, however, are interference-insensitive and lead to randomly distributed COs requiring the Mus81 and Mms4 proteins [4]. While several of the recombination intermediates produced during the recombination processes have been described, our understanding of the mechanisms governing the different pathways, as well as their putative interconnections, remain largely unraveled. A detailed study of a set of five *S. cerevisiae zmm* mutants *(mer3, msh5, zip1, zip2,* and *zip3)* demonstrated that the corresponding ZMM proteins are necessary for the correct progression from DSBs to stable single-end invasion (SEI) intermediates [5]. The biochemical functions of most of the actors are still under question, but recent data obtained on the Mer3 helicase [6] and on the Msh4/5 heterodimer [7] support the idea that the ZMM proteins bind to some early recombination intermediate to allow the formation of stable SEI intermediates, committing these to the interfering pathway. These multiple CO formation pathways do not coexist in all species [8], but the recent characterization of *Atmsh4* and *Atmer3* mutants showed the existence of two CO classes in *Arabidopsis,* with a major type being sensitive to interference and a minor interference-insensitive type [9–11].

Another important feature of the first meiotic prophase observed in the vast majority of organisms is the transitory setup, between homologous chromosomes, of a structure called the synaptonemal complex (SC) [12]. SC assembly starts with the formation of a single protein axis (called the axial element, AE) along each pair of sister chromatids. Then, while homologue recognition and recombination take place, the AEs of homologous chromosomes (then called the lateral element, LE) are closely connected together in a process called synapsis. The central element (CE) of the SC is polymerized creating a ladder-like structure holding each chromosome close to its homologue (for a review see [12–14]). A major component of the CE is a long coiled-coil protein (Zip1 in budding yeast, ZYP1 in *Arabidopsis,* SYCP1 in mammals, see [14]), whose polymerization forms the transverse filament. The way the mature SC actually forms is still unknown. A considerable amount of descriptive cytogenetic studies, however, has shown that there is a general tendency for early synapsis to occur at or near chromosome ends. Subsequently, additional synaptic initiation sites may occur interstitially, with the number of these sites being highly variable. For example, animals are known to have few of these, whereas higher plants have many [13]. Recent molecular data from budding yeast studies showed that the formation of a mature SC (i.e., polymerization of Zip1, formation of the CE) depends on a protein complex called the “synapsis initiation complex” (SIC) [15]. There are now several known components of the SIC [16–18] that all belong to the ZMM group of proteins. It appears that binding of Zip3 onto chromatin recruits both Zip2 and Zip4, which, in turn, induces Zip1 polymerization [17,18]. The biological function of these proteins is still poorly understood, but recent evidence suggests that they act on Zip1 polymerization through a pathway involving protein conjugation [19–21]. The fact that all the SIC components are necessary for class I COs, and that the number of SICs corresponds with the number of COs (see Discussion), strongly suggests that, at least in *S. cerevisiae,* synapsis proceeds from class I CO sites [22,23]. The existence of such SICs in other eukaryotes, as well as their possible link with CO precursors remains to be elucidated. Furthermore, unlike other ZMM proteins, Zip proteins are poorly conserved among species [21].

In this article, we report the characterization of a putative higher eukaryote SIC component: the *Arabidopsis* ZIP4 protein. Our data clearly indicate that AtZIP4 belongs to the ZMM pathway because it is necessary for class I CO setup. However, its requirement for CE polymerization is not observed since complete synapsis is achieved in *Atzip4* background.

## Results

### Identification and Molecular Characterization of the *Atzip4* Mutants

**Figure 1:**
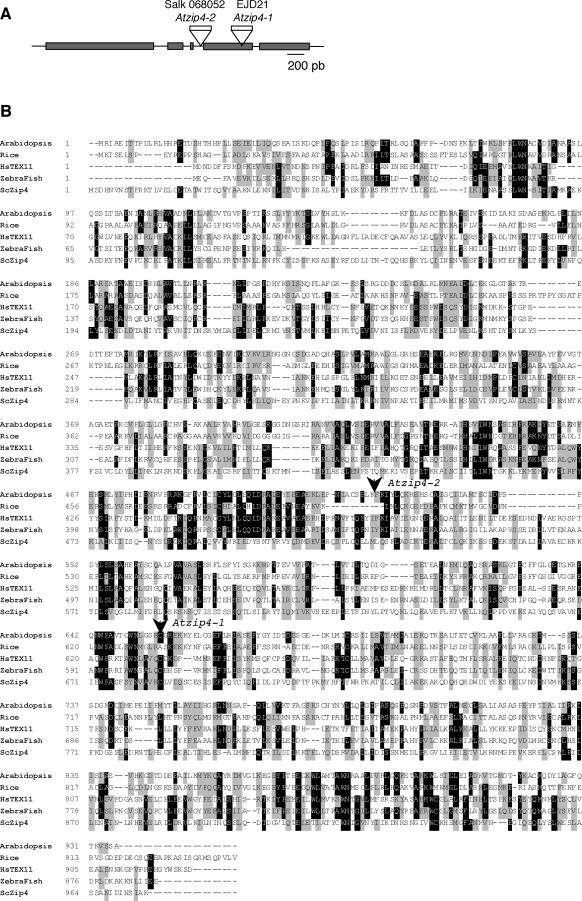
The Zip4 Family and *Atzip4* Mutations (A) Schematic representation of the *AtZIP4* coding sequence. Exons are represented as black boxes and T-DNA insertions in *Atzip4-1* and *Atzip4-2* alleles are indicated. (B) Alignment of *A. thaliana, S. cerevisiae, Homo sapiens, Oryza sativa,* and Dario reno Zip4 homologues. The numbers indicate aa positions; identical aas are boxed in black whereas similar aas are boxed in gray. The positions of the T-DNA insertions in the mutant alleles are indicated.

In a screen for A. thaliana T-DNA (Agrobacterium tumefaciens-transferred DNA) insertions that generate meiotic mutants (see Materials and Methods), we have isolated three allelic mutations corresponding to disruption of a predicted open reading frame of the *Arabidopsis* genome, At5g48390, annotated as a putative tetratricopeptide repeat–containing protein. The first two mutations correspond to insertion alleles (Figures 1 and S1), whereas the third mutation corresponds to a deletion allele in which no amplification of any part of At5g48390 could be detected (see Figure S1).

We isolated and sequenced the full-length At5g48390 cDNA from flower buds and found it encodes a 936-amino acid (aa) protein. Database searches using the BLASTP program (Blosum 45) for proteins similar to that encoded by At5g48390 produced the highest scores (outside the plant kingdom) with several mammalian sequences similar to a testis-specific expressed sequence (TEX11, 18% identity and 38% similarity over 894 aas) [24]. A second round of homology searches using TEX11 as the query revealed a significant similarity with the budding yeast Zip4/Spo22 protein (15% identity and 35% similarity over 532 aas, BLASTP, Blosum 45). A multiple sequence alignment of putative ScSpo22/Zip4 orthologues revealed an overall conservation of this protein between *S. cerevisiae,* vertebrates, and plants, with conserved residues throughout the entire length of the protein (Figure 1B) and largely exceeding the putative tetratricopeptide repeat domains (aa 134–167 and 443–517 found in PROSITE and Pfam databases). Reciprocally, iterate searches for putative ScZip4 homologues within the *Arabidopsis* genome using the PSI-BLAST program only picked up At5g48390 (19% identity and 35% similarity over 267 aas). These results led us to call the newly isolated gene *AtZIP4* and the corresponding mutations *Atzip4-1* and *Atzip4-2* (Figure 1A and 1B). The deletion allele (Figure S1) was called *Atzip4-3.*

Reverse-transcriptase PCR (RT-PCR) studies showed that *AtZIP4,* as with many *Arabidopsis* meiotic genes, is expressed at low level in roots and flower buds but not in leaves (unpublished data). Furthermore, RT-PCR studies on flower bud cDNA from mutant plants showed that wild-type transcript is not detected in *Atzip4-2,* and that a truncated form is expressed in *Atzip4-1* (Figure S1). Nevertheless, no phenotypic difference could be detected between both alleles, either between these two insertion mutants and the deletion allele *Atzip4-3,* suggesting that all of the three alleles correspond to null mutations, and that the partial *AtZIP4* cDNA expressed in *Atzip4-1* is not functional.

### The *Atzip4* Mutants Are Meiosis-Defective

**Figure 2:**
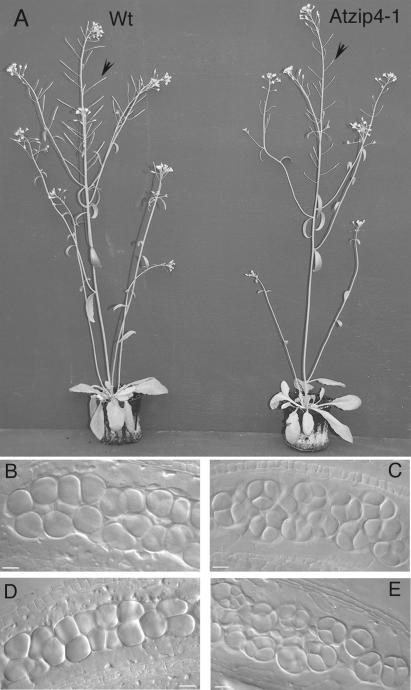
*Atzip4* Mutant Phenotype (A) Comparison of wild-type and homozygous *Atzip4-1* mutant plants after 30 d in the greenhouse. Arrows show siliques that elongate in wild type but not in mutant. (B–E) Male sporogenesis in wild type (B–C) and *Atzip4* mutant (D–E) shown for *Atzip4-1.* Male meiocytes (PMCs) are shown in (B and D) within the anthers, and the product of meiosis (tetrads or polyads) is shown in (C and E). Wt, wild type.

All three *Atzip4* mutants displayed the same phenotype: normal vegetative growth (Figure 2) but short siliques (Figure 2, arrows) suggesting fertility defects. The mean seed number per silique was 3.4 for *Atzip4-1* (*n* = 1,000) and 4.3 for *Atzip4-2* (*n* = 1,000), whereas wild-type siliques contained on average 63 and 71 seeds per silique for Ws (*Atzip4-1* ecotype) and Col-0 (*Atzip4-2* ecotype), respectively (*n* = 50).

We examined the reproductive development of these mutants and found that *Atzip4* are sterile due to abortion of male and female gametophytes (unpublished data). Comparison of the early stages of microsporogenesis revealed no difference between wild-type and mutant plants (Figure 2B and 2D): round pollen mother cells (PMCs) were found within the anther locules. In wild-type anthers, these cells underwent two meiotic divisions to produce a characteristic tetrad of microspores (Figure 2C). Meiosis products were also detected in mutant plants, but they lacked the regular tetrahedral structure and were either asymmetric tetrads or “polyads” containing more than four products (Figure 2E), suggesting that the meiotic program is disturbed in *Atzip4* mutants*.*

**Figure 3:**
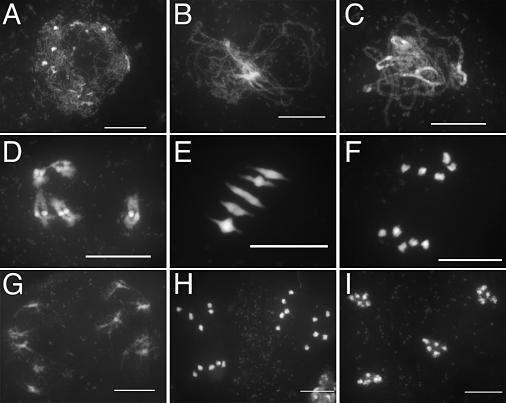
DAPI Staining of Wild-Type (Ws) PMCs during Meiosis (A) Leptotene, (B) zygotene, (C) pachytene, (D) diakinesis, (E) metaphase I, (F) end of anaphase I, (G) telophase I, (H) end of anaphase II, (I) end of meiosis. Bar, 10 μm.

We therefore investigated male meiosis by staining chromosomes with 4′,6-diamidino-2-phenylindole (DAPI). Wild-type *Arabidopsis* meiosis has been described in detail in [25], and the major stages are summarized in Figure 3. During prophase I (Figure 3A–3D), meiotic chromosomes condense, recombine, and undergo synapsis, resulting in the formation of five bivalents, each consisting of two homologous chromosomes attached to each other by sister chromatid cohesion and chiasmata, which become visible at diakinesis (Figure 3D). Synapsis (the close association of two chromosomes via an SC) begins at zygotene (Figure 3B) and is complete by pachytene, by which point the SC has polymerized along the whole length of the bivalents (Figure 3C). At metaphase I, the five bivalents are easily distinguishable (Figure 3E). During anaphase I, each chromosome separates from its homologue (Figure 3F), leading to the formation of dyads corresponding to two pools of five chromosomes (Figure 3G). The second meiotic division then separates the sister chromatids, generating four pools of five chromosomes (Figure 3H and 3I), which gives rise to tetrads of four microspores (Figure 2C).

**Figure 4:**
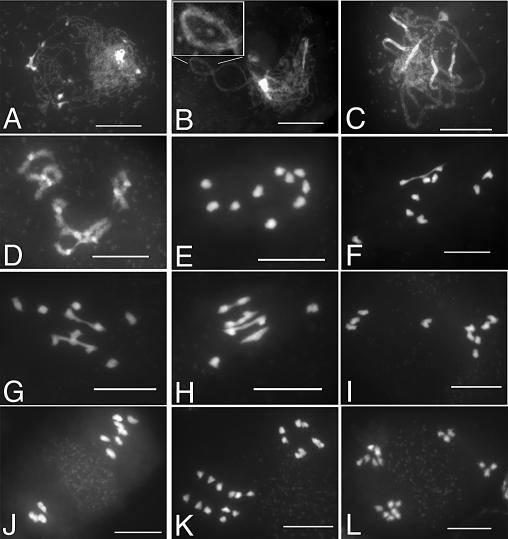
DAPI Staining of *Atzip4-1* PMCs during Meiosis (A) Leptotene, (B) zygotene: The synapsed portion of (B) has been magnified and is presented in the boxed area of the figure. (C) Pachytene, (D) diakinesis, (E–H) metaphase I, (I) anaphase I, (J) metaphase II, (K) anaphase II, (L) end of meiosis. Bar, 10 μm.

In *Atzip4* mutants*,* the early stages of meiosis could not be distinguished from wild type: chromosomes appeared as threads at leptotene (Figure 4A), condensed and synapsed (Figure 4B) until pachytene (Figure 4C). Aberrations, however, appeared at early diakinesis in *Atzip4* mutants, with cells showing a mixture of bivalents and univalents (Figure 4D). At metaphase I, this defect became even more obvious with mutant cells showing a variable number of bivalents (from 0 to 4, Figure 4E–4H). During subsequent anaphase I, random segregation of the chromosomes was observed in the mutant (Figure 4I and 4J). Then, in the second meiotic division, sister chromatids segregated normally (Figure 4K), giving rise to a variable number of daughter cells containing aberrant numbers of chromosomes (compare 3I to 4L).

An analysis of female meiosis in *Atzip4* identified defects similar to those seen during male meiosis (unpublished data). Thus, *AtZIP4* is involved in both male and female meiosis, and its disruption leads to a decrease in the number of bivalents.

### *AtZIP4* Is Necessary for CO Formation

The level of meiotic recombination in *Atzip4* mutants was estimated by two independent methods. First, the overall level of meiotic recombination was estimated by measuring the mean number of chiasmata at metaphase I on spread PMC chromosomes, as described in [26]. The *Atzip4* alleles are in two different ecotypes (Ws for *Atzip4-1* and *Atzip4-3* and Col-0 for *Atzip4-2*), and because this measurement is known to vary among genotypes [27], we compared chiasma frequency in all *Atzip4* mutants to their respective wild type. As shown in Table 1, we observed a strong decrease in chiasma formation in every mutant in comparison to wild types: 7.7 times less in *Atzip4-1,* 5.6 times less in *Atzip4-2,* and 9 times less in *Atzip4-3,* corresponding to a residual level of chiasma of 12.9% for *Atzip4-1,* 17.7% for *Atzip4-2,* and 10.8% for *Atzip4-3,* respectively. Statistical analyses on these data showed that if the mean chiasma number is different between Ws and Col-0 (*t* test, *p* = 3.93216 10^−15^) and between all mutant and their respective wild types, there is no difference between the two *Atzip4* alleles in Ws ecotype (*Atzip4-1* and *Atzip4-3, p* = 0.40, *t* test).

**Table 1:**
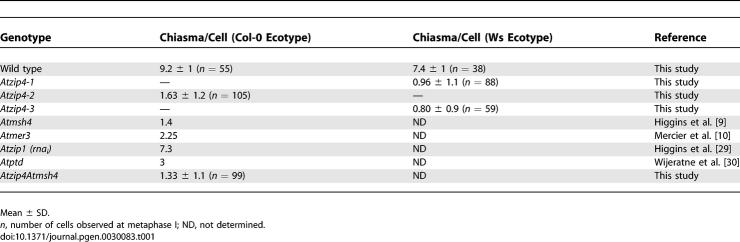
Chiasma Frequency in Male Meiosis

Secondly, the level of recombination was calculated genetically on several intervals of Chromosome I (Table 2). Taking advantage of the mutants' different backgrounds, we crossed a heterozygous *Atzip4-1^+/−^* (Ws) plant with an *Atzip4-2^+/−^* (Col-0) plant and in the F1 generation selected either *Atzip4* mutants *(Atzip4-1Atzip4-2)* or homozygous wild-type plants. In order to measure male meiosis recombination rates in wild-type and *Atzip4* backgrounds, we then performed backcrosses between these lines and the Col-0 ecotype used as female. We chose microsatellite markers polymorphic between the two ecotypes to measure the percentage of recombination in the two genotypes (wild type or *Atzip4* mutant) in this hybrid background Ws/Col-0 (Table 2). For the three intervals tested, we found that the level of meiotic recombination decreased by a factor of approximately 5 (from 4 to 6) in the mutant background compared to wild type. Chiasma frequency in this hybrid background was also measured and found to be identical to the observed frequency in Ws background (7.6 ± 0.9, *n* = 37) for the wild-type Ws/Col-0 and 0.92 ± 0.8 (*n* = 105) for the *Atzip4* mutant.

**Table 2:**
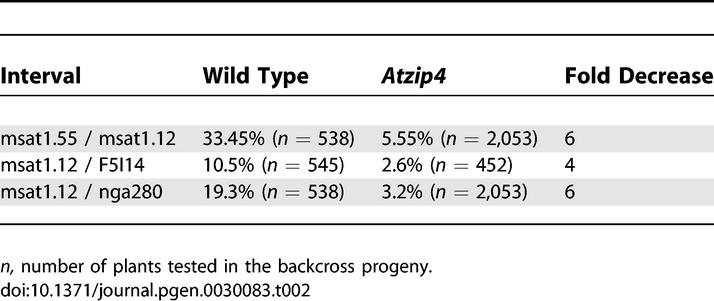
Recombination Rate in Male Meiosis

These experiments demonstrate that the shortage of bivalents observed at metaphase I in *Atzip4* mutants reflects a general reduction in CO formation. The decrease in recombination frequency varied from 5- to 9-fold depending on the method used, the allele tested, or the genetic background (Tables 1 and 2).

The phenotype of *Atzip4* mutants is similar to that of other recently described *zmm Arabidopsis* mutants, and specifically, *Atzip4* mutants show a reduction in CO frequency comparable to that of the *Atmsh4* mutant (Table 1). Thus, in order to check whether these genes belong to the same epistasis group, we quantified the level of remaining COs in the *Atmsh4Atzip4* double mutant (see Materials and Methods). We did not observe a significant decrease in the mean number of chiasmata per PMCs between *Atzip4* and double *Atmsh4Atzip4* (*t* test, *p* > 0.05), showing that these two genes function in the same pathway of CO formation.

### Recombination Dynamics, but Probably Not Recombination Initiation, May Be Modified in *Atzip4* Mutants

**Figure 5:**
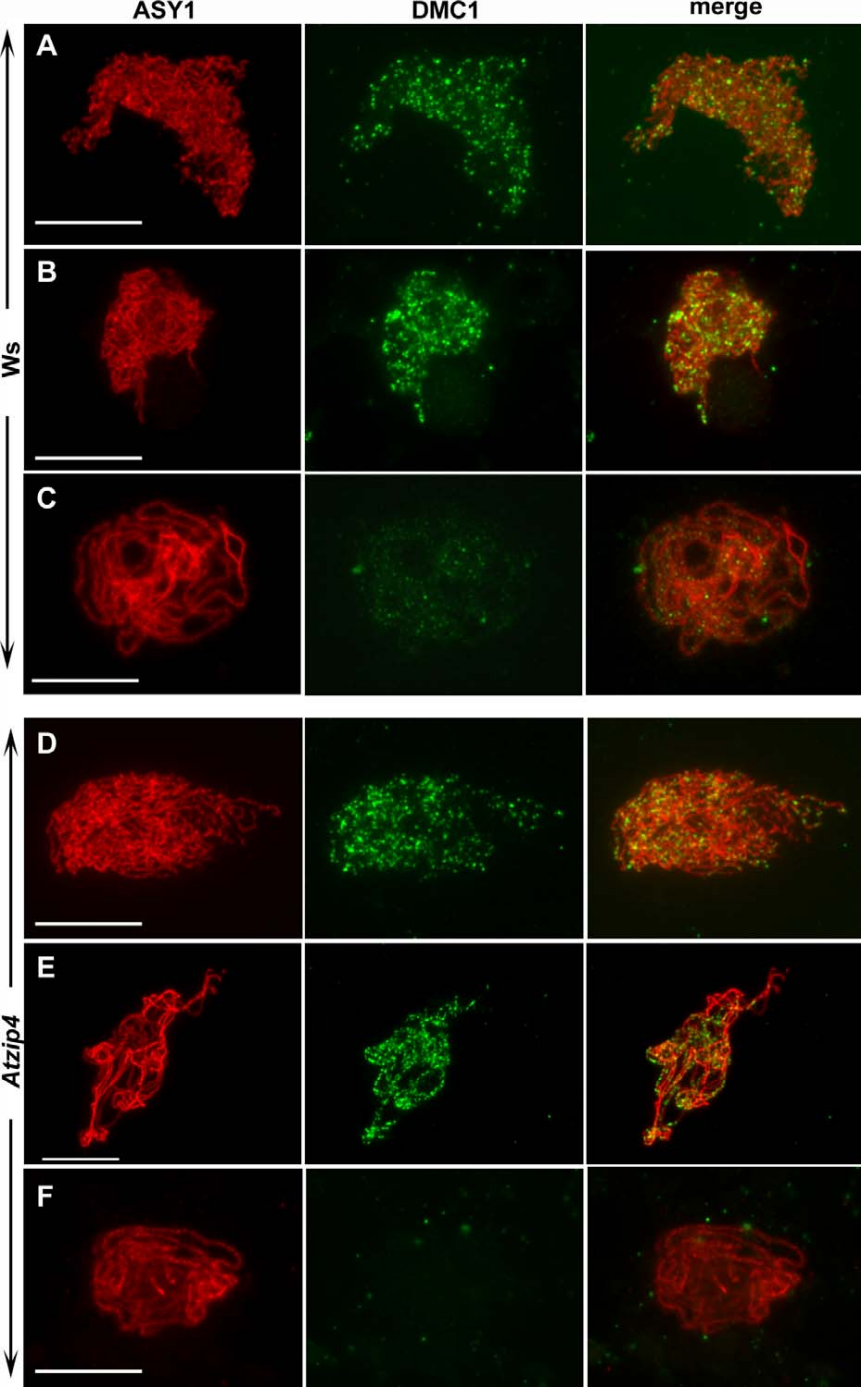
Coimmunolocalization of ASY1 and AtDMC1 in Wild-Type and Mutant Meiocytes Coimmunolocalization of ASY1 (red) and DMC1 (green) in wild-type (Ws) and *Atzip4–1 (Atzip4)* PMCs. For each cell, each single staining is shown as well as the overlay of both signals (merge). (A and D) Leptotene, (B and E) zygotene, (C and F) pachytene. Bar, 10 μm.

Because *Atzip4* mutants display a strong decrease in CO frequency, we wondered whether the early stages of meiotic recombination were disrupted. We therefore analyzed the nuclear distribution of the DMC1 protein, which is an essential component of the recombination machinery. Its appearance on meiotic chromosomes during prophase is thought to reflect the progression of recombination repair. To date, DMC1 staining on meiotic chromosomes in plant cells has not been described. We therefore designed an antibody directed against a synthetic peptide specific to the DMC1 protein (no signal in a *dmc1* background, see Materials and Methods). To accurately define the temporal distribution of DMC1 throughout meiosis, immunolocalization studies were carried out by double-labeling wild-type and mutant PMCs with anti-DMC1 and anti-ASY1 (a protein associated with the AE of the SC, [28]) antibodies. In wild-type cells, DMC1 foci appeared at mid-leptotene, when ASY1 was detectable as continuous stretches (Figure 5A) and persisted during zygotene when ASY1 localized to full-length chromosome axes and the chromosomes started to synapse (Figure 5B). Quantification of the number of foci observed at these stages showed a mean number of foci in Ws PMCs of 235 ± 84 (*n* = 43). DMC1 staining then tended to disappear, and only a few residual DMC1 foci could be seen by the pachytene stage (Figure 5C). A similar pattern of DMC1 labeling was observed in *Atzip4-1,* with strong fluorescence from mid-leptotene to zygotene, and reduction in the number of foci while synapsis proceeded, until finally at pachytene fluorescent foci were no longer visible (Figure 5D–5F). Nevertheless, quantification of the DMC1 signal (all stages taken together) showed that the mean number of foci observed in the mutant background was slightly but significantly (*t* test, *p* = 0.002) higher than in wild type, with a mean number of foci of approximately 294 ± 75 (*n* = 46). This suggests that in *Atzip4* the dynamics of DSB formation and/or repair may be slightly modified, but excludes the possibility that the shortage in chiasmata is a consequence of an overall decrease in meiotic recombination events.

### Remaining COs in *Atzip4* Are Not Subject to Interference

We tested for interference by comparing the number of single and double COs in two adjacent intervals of Chromosome I (Table 3). In wild type, chromosomes that recombined in interval I showed a strong decrease in the frequency of COs in adjacent interval II (7.2% recombination in II when COs have occurred in I to be compared to 19.3% without preselection of recombinants in interval I) and vice versa (12.5% compared to 33.45%). This can be expressed by the coefficient of coincidence (CC) defined as the proportion of observed double COs divided by the expected proportion of double COs if they were independent (that is, the product of each individual CO frequency) [2]. When COs in the two intervals are independent the CC is 1, whereas the stronger the interference between two adjacent COs, the lower the CC. As shown in Table 3, the wild-type CC for the two intervals was 0.37. Statistics performed on this data showed that in wild type the proportion of single and double COs deviates highly significantly from that expected without interference (χ^2^ = 25.4, *p* < 0.001). In the *Atzip4* background, we found a CC close to 1 (Table 3), and statistical analyses of these results confirmed that the proportion of double CO was very close to that predicted without interference (χ_(1)_^2 ^ = 0.046, *p* > 0.1). On the contrary, we found that the results obtained in *Atzip4* are significantly different (χ_(1)_^2 ^ = 5.79, *p* < 0.025) from the expected distribution obtained by applying the wild-type CC of 0.37. Thus, the COs occurring in these two adjacent intervals in *Atzip4* are not sensitive to interference.

**Table 3:**
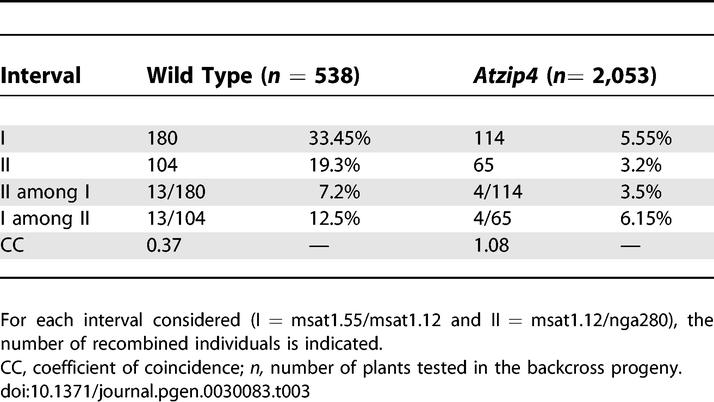
CO Interference between Two Chromosome I Intervals

### Synapsis Occurs in the Absence of AtZIP4

**Figure 6:**
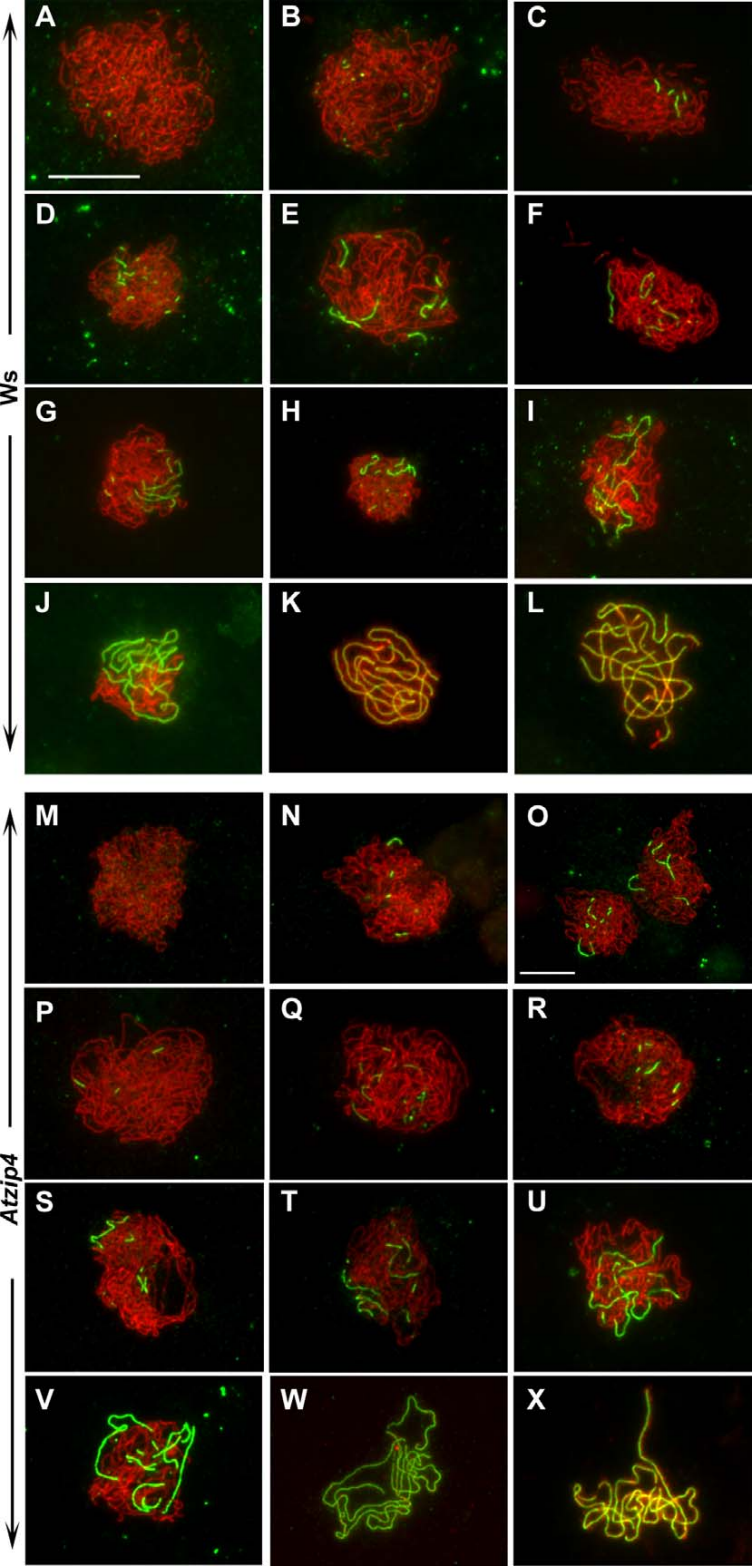
Coimmunolocalization of ASY1 (Red) and ZYP1 (Green) in Wild-Type (Ws) and Mutant *(Atzip4)* PMCs Prophase I cells showing increasing level of synapsis (according to anti-ZYP1 labeling) are shown for both genotypes: absence of synapsis (leptotene, A and M), partial synapsis (zygotene, B–J and N–V), and full synapsis (pachytene, K–L and W–X). For all cells, only the merge signal is shown, but Figures S2 and S3 provide each single staining. Bar, 10 μm (bar presented on A applies for all cells except O).

From observations of DAPI-stained preparations, chromosomes in *Atzip4* mutants appeared to be able to synapse (compare Figure 3C to 4C). To fully understand the effect of the *Atzip4* mutations on SC formation, meiotic chromosomes were immunolabeled with antibodies against ASY1 and ZYP1 (a major component of the CE of the SC, [29]). There was no obvious difference in mutant compared to wild-type cells (Figure 6). Briefly, we observed axial staining with ASY1 that commenced during early prophase and became visible as threads while leptotene progressed (Ws, Figure 6A; *Atzip4-1,* Figure 6M). ZYP1 appeared very early on chromosomes (defining the beginning of zygotene stage, that is, the beginning of synapsis) as foci that quickly elongated yielding a mixture of foci and short stretches of ZYP1 labeling (Figure 6B–6H for wild type; Figure 6N–6T for *Atzip4-1*). The number of these first ZYP1 sites varied from one to more than 20. Synapsis then progressed very asynchronously with some bivalents completing synapsis before others had hardly started (Figure 6I and 6J for wild type and Figure 6U and 6V for *Atzip4-1*). Finally, complete synapsis was observed in both genotypes (Figure 6K and 6L for wild type and Figure 6W and 6X for *Atzip4-1*). In order to detect possible differences in synapsis efficiency between mutant and wild type, we measured the proportion of cells showing full synapsis (pachytene stage, Figure 6K–6L and 6W–6X) among the group of cells that were immunolabeled by anti-ZYP1 in wild type and mutant. These proportions were found not to be statistically different (χ_(1)_^2^ = 2.15, *p* > 0.14) with 44.8% of full pachytene for wild type (Ws, *n* = 174) versus 37.8% for mutant cells (*Atzip4-3, n* = 262). We also measured the total length of the SC at pachytene and found that it was the same in both genotypes: 154 ± 31 μm in wild type (Ws, *n* = 15) and 153 ± 23 μm in *Atzip4-1* (*n* = 23). Therefore, AtZIP4 is not required for synapsis to proceed.

We quantified early synapsis events by counting the number of ZYP1 stretches in early to mid-zygotene, that is, as soon as ZYP1 labeling can be detected on chromosomes and until it covers less than 40% of the nuclei that correspond to nuclei of Figure 6B–6H and 6N–6T. We found that the mean number of synapsis sites per nucleus was significantly different between *Atzip4-1* and wild-type ecotype Ws (*t* test, *p* < 0.001). For the wild type the mean number of ZYP1 stretches was 7.6 ± 3.2 (mean ± SD, *n* = 43), whereas it was only 4.9 ± 2.8 (*n* = 39) for *Atzip4-1.* Therefore, the *Atzip4* mutation may not prevent full synapsis from occurring, but it can modify synapsis initiation and/or synapsis dynamics.

## Discussion

In *S. cerevisiae,* Zip4/Spo22 was identified as a member of the ZMM group of proteins that also includes Zip1, Zip2, Zip3, Msh4, Msh5, and Mer3 and together control the formation of class I COs [3]. In budding yeast, all ZMM proteins are also required for normal synapsis, and Zip4 was recently shown to be a central protein of the SICs, from which the polymerization of the transverse filament proceeds. These different aspects of Zip4 function will be discussed in light of our results on the *Arabidopsis* ZIP4 protein.

### AtZIP4 Is Necessary for Class I CO Formation

Recent studies suggest that like *S. cerevisiae, Arabidopsis* possesses at least two CO pathways. The major (class I) pathway depends on *AtMSH4* [9], *AtMSH5* (F. C. H. Franklin and R. Mercier, personal communication), *AtMER3* [10,11], and possibly a newly identified gene called *PTD* [30]. We show here that *AtZIP4* is likely to be another key player in this pathway.

First, we showed that the AtZIP4 protein is necessary for 85% of the meiotic COs in *Arabidopsis,* as are AtMSH4 [9] and AtMSH5 (F. C. H. Franklin and R. Mercier, personal communication), and we demonstrated that *AtMSH4* and *AtZIP4* belong to the same epistasis group, with regards to their effect on CO level. Secondly, the ZMM proteins are thought to specifically drive the formation of class I but not class II COs. Accordingly, the remaining COs observed in the *zip1, mer3,* and *msh4* budding yeast mutants no longer display interference [18,31–33] and those in *zip4* display negative interference [18]. In the case of *Atzip4,* genetic analysis using two sets of adjacent markers on Chromosome I showed that the occurrence of remaining COs in this part of the genome was not subjected to interference. This situation seems to be the same for the other *Arabidopsis* ZMM proteins identified so far: the remaining COs in *ptd* and *Atmsh4* are randomly distributed among cells, consistent with an absence of interference by one CO on another [9,30], and genetic analysis showed that the occurrence of remaining COs in *Atmer3* did not display interference [10]. Lastly, the study of early recombination events in *Atzip4,* by immunolabeling with anti-DMC1, demonstrated that even if the dynamics of DSB repair are modified in the *Atzip4* background, the CO defects observed do not reflect an overall decrease in recombination events. The same results have been reported for *Atmer3* [10] and *Atmsh4* [9], showing that all these mutants are defective in the maturation of recombination events leading to class I CO formation. Therefore, we can conclude that AtZIP4 possesses all the characteristics of a ZMM protein.

As suggested by our observations of DMC1 foci, it seems highly likely that in the *zmm* mutant backgrounds, recombination is initiated at the wild-type level, but that CO maturation is prevented. Because chromosome fragmentation was never observed in any of the Arabidopsis zmm mutants (this study or [9,10], for example), DSB repair appears to still take place. Unfortunately, we cannot decipher which repair pathway is in use (repair onto the homologous chromosomes yielding noncrossover products or onto the sister chromatid, for example). These data are consistent with data from budding yeast *zmm* mutants in which no modification in DSB levels was observed [5,32], but DSB repair was affected at steps yielding stable SEI molecules [5]. *zmm* mutants (and *zip4* in particular) show decreased CO formation without noncrossover increase [5,16,18], and most DSBs disappeared because they were either degraded or repaired through nonconventional pathways [5].

### Synapsis Completion Is Normal in the Absence of AtZIP4

In budding yeast, formation of the SC depends on a protein complex called the SIC [15]. So far, Zip2, Zip3, and Zip4 are the known key components of the SIC. These three proteins are not necessary for initial binding of Zip1 to chromosomes but are necessary for the progression of synapsis [18]. Zip2/Zip4 are thought to play vital roles in synapsis initiation [18], whereas the function of Zip3 might be to stabilize the Zip2/Zip4 complex onto chromosomes. The Zip proteins are poorly conserved among eukaryotes [21], but a Caenorhabditis elegans Zip3 orthologue was described recently [34]. Synapsis proceeded normally in the *C. elegans zhp-3* mutant even though CO formation was defective. Nevertheless, unlike meiosis in most organisms (see below), synapsis in C. elegans is totally uncoupled from recombination; therefore, generalizations regarding this apparent divergence in SIC function as it may apply to other higher eukaryotes are hard to make. Our results on another core component of the SICs (Zip4) show that in A. thaliana the role of Zip proteins as SIC components is also not conserved (not only normal pachytene stages are achieved in *Atzip4* mutants, but these occur at wild-type frequency), while synapsis is indeed dependent on recombination in *Arabidopsis* [35]. Therefore, the two aspects of Zip4 function, recombination control and synapsis setup, can be uncoupled. Nevertheless, we cannot exclude a role, direct or not, of AtZIP4 in early synapsis since we observed a 35% decrease of early ZYP1 foci number in *Atzip4* mutants. This diminution could reflect either a global diminution of the numbers of sites from which Zip1 polymerization proceeds, or, alternatively, it might reflect a perturbation of synapsis dynamic in *Atzip4* mutants.

### What Is the Link between Synapsis and CO Sites?

Data obtained in budding yeast suggest that most, if not all, SC initiation sites correspond to CO sites. Indeed, good correlations were observed between the number of COs and the number of Zip3 foci. For example, when the CO frequency decreased in leaky *spo11* mutants, [36] observed a correlation between the amount of Zip3 foci, SC formation, and CO level. Reciprocally, the increased CO frequency observed in a *sgs1* mutant was accompanied by an increase in Zip3 foci [37]. Therefore, the idea emerged that CO intermediates (and only those leading to class I COs) provide the sites for Zip1 nucleation [1,22,23].

The situation is less clear in other organisms. In many species synapsis progression was investigated by counting SC stretches on silver-stained early zygotene chromosomes. Even if this technique probably underestimates the number of SC initiation sites, it gives an idea of the synapsis initiation pattern. Synapsis appears to commonly take place at least at the terminal or subterminal region of chromosomes. Furthermore, a variable number of interstitial initiation sites has also been observed [13,38]. Plants are known to have high numbers of such synapsis initiation sites (for example, 76 SC segments/nucleus in rye [39], up to 300 in *Tradescantia* [40], and up to 36 for a single lily bivalent [41]). The average chiasmata number per bivalent, however, is much lower and hardly varies, at around 1–3 per bivalent (2.45 per bivalent for the lily, for example). Animals have much lower numbers of interstitial pairing sites [42], but even so, the ratio between synapsis initiation sites and chiasmata can be higher than 1 [43]. Lastly, immunocytology studies on mouse showed that in mammals, synapsis can proceed from sites different than CO sites (cited in [14]).

In this study, we show that when 85% of COs (which probably represent all the class I COs) are suppressed in *Arabidopsis,* synapsis is not prevented. More precisely, in *Atzip4* mutants we observed an average of one chiasma per meiocyte, whereas the five chromosome pairs still synapsed at pachytene, showing that the absence of CO within a bivalent does not prevent synapsis from occurring. More generally, only mild synapsis defects were reported for the other Arabidopsis zmm mutants [10,11,30]. In the case of *Atmsh4,* it has been shown that prophase I is delayed compared to wild type (from 30 to 38 h) suggesting that the timing of synapsis could be modified when an *Arabidopsis* ZMM is not functional [9]. In the case of *Atzip4* mutants, the observation that the average number of synapsis tracks was significantly lower than in wild type suggests that if CO I intermediates are not absolutely required for synapsis in plants they may correspond to some of the synapsis initiation sites. This could explain why examples of correlation between synapsis initiation sites and CO sites were reported in plants, such as in chromosomal inversions of maize (see [44], for example).

In conclusion, our results show that, in *Arabidopsis,* synapsis either does not depend on CO I, or depends upon CO I precursors upstream of the ZMM proteins. Furthermore, in *Arabidopsis,* synapsis initiation sites may coincide with sites of future CO formation, but this does not appear to be a unique or indispensable relationship.

### What Is the Link between Synapsis and Recombination?

We have shown that CO I intermediates are not necessary for synapsis in *Arabidopsis;* nevertheless, it is clear that recombination and synapsis remain strongly connected in most higher eukaryotes. Drosophila melanogaster and C. elegans are the only organisms in which the two processes are uncoupled since both can form normal SCs in the absence of any recombination [45,46]. To date, these appear to be exceptions since all the other organisms studied *(A. thaliana,* mouse, yeast, *coprinus)* are asynaptic when DSBs are prevented [47]. Ultrastructural studies performed on different plant species showed that synapsis proceeded from sites of AE interaction (axial associations) that load early recombination nodules (RAD51-containing nodules), establishing the link between recombination and synapsis [48–51]. In *Arabidopsis,* these nodules have not been described because of the difficulties involved in preparing chromosomes for electron microscopy in this species. Nevertheless, in *Arabidopsis,* the early association of ZYP1 with chromosomes in foci and its subsequent extension was shown to depend on AtSPO11–1 and AtDMC1, respectively [29], showing that normal synapsis is dependent upon early recombination intermediates as in other species. Nevertheless, close to 250 early recombination intermediates can be observed in *Arabidopsis* (as estimated by the number of RAD51/DMC1 foci), whereas the number of ZYP1 initial foci does not exceed 20 (this study and [29]). Thus, it appears that only a minority of these early recombination intermediates are actually acting as synapsis initiation sites. The way these are selected, as well as the specific components of synapsis initiation complexes in higher eukaryote, remains to be elucidated.

## Materials and Methods

### Plant material.

The *Atzip4-1* mutant (EJD21 line) and *Atzip4-3* (EFS349 line) were obtained from the Versailles *Arabidopsis* T-DNA transformant collection [52]. Mutant screening was performed as described in [53]. The *Atzip4-2* mutant, line Salk_068052, was obtained from the collection of T-DNA mutants at the Salk Institute Genomic Analysis Laboratory (SIGnAL, http://signal.salk.edu/cgi-bin/tdnaexpress) [54] and provided by the Nottingham *Arabidopsis* Stock Centre (NASC) (http://nasc.nott.ac.uk). The *Atmsh4* mutant corresponds to line Salk_136296 and was described in [9].

### Growth conditions.

*Arabidopsis* plants were cultivated in a greenhouse or growth chamber under the following conditions: photoperiod 16 h/day and 8 h/night; temperature 20 °C day and night; humidity 70%.

### Genetic analyses.

Isolation of *Atzip4-1:* the EJD21 line segregated 3:1 for the meiotic mutation (revealing the presence of a single recessive mutation) and 15:1 for kanamycin resistance (one of the T-DNA markers), suggesting the presence of at least two inserts. After crossing to wild type, linkage between a single T-DNA insert and the meiotic phenotype was checked as described in [35].

We tested for allelism between the *Atzip4-1* and *Atzip4-2* mutations by crossing *Atzip4-1^−/+^* and *Atzip4-2^−/+^*. Among the F1 plants, one-fourth was semi-sterile and possessed each of the mutant alleles.

Double mutants for *Atmsh4* and *Atzip4-2* were obtained by crossing plants heterozygous for each mutation. The resulting hybrids were self-pollinated. We used PCR screening to select the sterile plants in the F2 progeny homozygous for both mutations.

Recombination rates and interference study: Plants heterozygous for *Atzip4-1* mutation (Ws ecotype) were crossed to heterozygous plants for *Atzip4-2* (Col-0 ecotype). F1 plants, either homozygous semi-sterile *Atzip4-1/Atzip4-2* or homozygous fertile *AtZIP4^+/+^*, were selected after PCR genotyping and crossed onto a wild-type Col-0 plant. Progeny were sown in vitro and genotyped for several loci on Chromosome I with microsatellite markers showing polymorphisms between the two ecotypes Ws and Col-0: msat1.55, msat1.12, F5I14, and nga280 ([55] and http://www.inra.fr/vast/msat.php). For interference studies, plants showing recombined chromosomes in interval I (msat1.55 and msat1.12) and/or in adjacent interval II (msat1.12 and nga280) were scored as well as the plants that have not recombined in one or the other of the interval. We tested for deviation from an expected repartition with or without interference by means of a Chi-squared (χ^2^) test, applying a degree of freedom of 1.

### Molecular biology.

Isolation of plant T-DNA flanking sequences: The right border of the T-DNA insert of *Atzip4-1* was isolated using kanamycin rescue experiments, according to [56]. The left border of T-DNA insert in *Atzip4-1* was PCR amplified (P4 and LbBar2) and subsequently sequenced, showing the T-DNA was inserted in a predicted open reading frame of the *Arabidopsis* genome, At5g48390.

Sequencing of *AtZIP4* cDNA: cDNA synthesis was performed with Superscript RT (Invitrogen, http://www.invitrogen.com) from total RNA (3 μg) extracted from Ws young flower buds. 3′ RACE experiments were performed using Invitrogen system for Rapid Amplification of cDNA Ends, version 2.0. For 3′ RACE experiments specific primers used were P3: GGGTCAAGGTGTGGGAAGGA and P8: GTGGTGAATTCTTGAGGCTGGC. RACE products were cloned into pCR2.1-TOPO (Invitrogen) and sequenced.

Oligonucleotides for PCR genotyping: The right border of the *Atzip4-1* T-DNA was amplified by PCR with primers P4: CCGTGTATGTCATACGCAAGT and TAG3:CTGATACCAGACGTTGCCCGCATAA; the left border was amplified with P3: GGGTCAAGGTGTGGGAAGGA and LbBar2: CGTGTGCCAGGTGCCCACGGAATAG. Wild-type *AtZIP4* was amplified with primers P3 and P10: CCAACCCGATGCTCAGCCA. For *Atzip4-2,* oligonucleotides P3R: TCCTTCCCACACCTTGACCC and P5: GACTGCTGGAGCAGAAACT were used for the wild-type allele and P3R with LbSALK2: GCTTTCTTCCCTTCCTTTCTC for the mutant allele. *AtMSH4* wild-type allele was amplified using primers 636296U: CTTCTTGCAGGTTGTGTTTG and 636296L: GCCAGCTGTTTTTGTTGTC and mutant allele using 636296L and LbSalk2.

### Sequence analyses.

Protein sequence similarity searches were performed at the National Center for Biotechnology Information (http://www.ncbi.nlm.nih.gov/BLAST) and at the *Arabidopsis* Information Resource (TAIR, http://www.arabidopsis.org/Blast), using BLOSUM45 matrix and default parameters. Sequence analyses were performed with BioEdit software (http://www.mbio.ncsu.edu/BioEdit/bioedit.html).

### Antibodies.

The anti-ASY1 polyclonal antibody has been described elsewhere [28]. It was used at a dilution of 1:500. The anti-ZYP1 polyclonal antibody was described by [9]. It was used at a dilution of 1:500.

The anti-DMC1 antibody was obtained by immunizing a rabbit with a synthetic peptide conjugated with KLH (Eurogentec, http://www.eurogentec.com). The synthetic peptide consisted of 18-aa residues from positions 1 to 18 of the *Arabidopsis* DMC1 protein (mmaslkaeetsqmqlver) and was designed so that the anti-DMC1 antibodies would specifically recognize AtDMC1 and not cross-hybridize with AtRAD51. Rabbit anti-DMC1 antibodies were purified as described in [57]. Specificity of the purified anti-DMC1 antibodies was checked by including the *dmc1* mutant [58] as a negative control in immunolocalization experiments. The working dilution of the purified serum for cytology was 1:20.

### Microscopy.

Comparison of early stages of microsporogenesis and the development of PMCs was carried out as described in [35]. Preparation of prophase stage spreads for immunocytology was performed according to [28] with the modifications described in [59].

All observations were made using a Leica (http://www.leica.com) DM RXA2 microscope; photographs were taken using a CoolSNAP HQ (Roper, http://www.roperscientific.com) camera driven by OpenLAB 4.0.4 software; all images were further processed with OpenLAB 4.0.4 or AdobePhotoshop 7.0 (http://www.adobe.com). SC length measurement was performed using Optimas (Bioscan Incorporated, http://www.bioscan.com) software.

## Supporting Information

Figure S1 Molecular Characterization of the *Atzip4* Alleles (A) Localization of the primers used for *Atzip4* molecular characterization.
(B) RT-PCR on *Atzip4-1* and *Atzip4-2* alleles.
RT-PCR on cDNA isolated from flower buds from *Atzip4-1* (lane 1), *Atzip4-2* (lane 2), or wild-type (lane 3) plants. L: Fermentas 1-kb DNA ladder. (B1) For *AtZIP4* amplification, a nested PCR was performed, first with primers P5 and P13, second with primers P12 and P14. Expected amplification size for wild-type cDNA sample: 250 bp; for genomic amplification: 430 bp. (B2) cDNA was calibrated according to the expression of the adenine phosphoribosyltransferase-encoding gene (*APT,* [60])
(C) PCR characterization of the *Atzip4-3* allele.
Genomic DNA amplification was performed using a series of primer combination covering the whole *AtZIP4* coding sequence (see Figure S1A) or using a set of primers amplifying the control *APT* gene. (1) DNA from a fertile heterozygous *Atzip4-3^+/−^* plant*.* (2) DNA from a sterile mutant *Atzip4-3^−/−^* plant. (3) DNA from a wild-type plant. (4) Water. Not shown: PCR with P18 and P22 showing also no amplification on mutant DNA.
(314 KB DOC)

Figure S2 Single Color Images of Figure 6A–6L Coimmunolocalization of ASY1 (red) and ZYP1 (green) in wild-type PMCs. Prophase I cells showing increasing level of synapsis (according to anti-ZYP1 labeling) are shown: absence of synapsis (leptotene, A), partial synapsis (zygotene, B–J), and full synapsis (pachytene, K–L). For each cell, single labeling is shown as well as the merge signal. Bar, 10 μm.
(2.2 MB TIF)

Figure S3 Single Color Images of Figure 6M–6X Coimmunolocalization of ASY1 (red) and ZYP1 (green) in *Atzip4-1* PMCs. Prophase I cells showing increasing level of synapsis (according to anti-ZYP1 labeling) are shown: absence of synapsis (leptotene, M), partial synapsis (zygotene, N–V), and full synapsis (pachytene, W–X). For each cell, single labeling is shown as well as the merge signal. Bar, 10 μm.
(2.2 MB TIF)

### Accession Numbers

GenBank (http://www.ncbi.nlm.nih.gov/Genbank) accession number for AtZIP4 cDNA is EF176583. The accession number for HsTEX11 is AAH36016, Rice NP_915110, and Zebrafish XP_692604. The accession number for the budding yeast Zip4/Spo22 protein is NP_012192.

## Abbreviations

aa: amino acid
AE: axial element
CC: coefficient of coincidence
CE: central element
CO: crossover
DSB: double-strand break
LE: lateral element
PMC: pollen mother cell
RT-PCR: reverse-transcriptase PCR
SC: synaptonemal complex
SEI: single-end invasion
SIC: synapsis initiation complex
T-DNA: *Agrobacterium tumefaciens-*transferred DNA
