# Supplementary material for: Zip4/Spo22 Is Required for Class I CO Formation but Not for Synapsis Completion in Arabidopsis thaliana
Source: PLoS Genet. 2007 May 25;3(5):e83. doi: 10.1371/journal.pgen.0030083 (PMC1877879; doi:10.1371/journal.pgen.0030083)
Supplement: Figure S1 — (A) Localization of the primers used for Atzip4 molecular characterization. (B) RT-PCR on Atzip4-1 and Atzip4-2 alleles. RT-PCR on cDNA isolated from flower buds from Atzip4-1 (lane 1), Atzip4-2 (lane 2), or wild-type (lane 3) plants. L: Fermentas 1-kb DNA ladder. (B1) For AtZIP4 amplification, a nested PCR was performed, first with primers P5 and P13, second with primers P12 and P14. Expected amplification size for wild-type cDNA sample: 250 bp; for genomic amplification: 430 bp. (B2) cDNA was calibrated according to the expression of the adenine phosphoribosyltransferase-encoding gene (APT, [60]) (C) PCR characterization of the Atzip4-3 allele. Genomic DNA amplification was performed using a series of primer combination covering the whole AtZIP4 coding sequence (see Figure S1A) or using a set of primers amplifying the control APT gene. (1) DNA from a fertile heterozygous Atzip4-3+/− plant. (2) DNA from a sterile mutant Atzip4-3−/− plant. (3) DNA from a wild-type plant. (4) Water. Not shown: PCR with P18 and P22 showing also no amplification on mutant DNA. (314 KB DOC) [file pgen.0030083.sg001.doc]

**Figure S1: Molecular characterisation of the *Atzip4* alleles**

## A: Localisation of the primers used for *Atzip4* molecular characterisation

### Atzip4-2

### Atzip4-1

# P13

**P4**

**P17**

**P7**

**P22**

# P14

**P5**

**P12**

**P3**

**P3R**

**P6**

**P18**

Primer sequences:

P3: GGGTCAAGGTGTGGGAAGGA

P3R: TCCTTCCCACACCTTGACCC

P4: CCGTGTATGTCATACGCAAGT

P5: GACTGCTGGAGCAGAAACT

P6: GCGAACTGCTATCAGTACCA

P7: TCGCAATGTTCTCGGCGGA

P12: GAAGGTGTCATGTCAGTGC

P13: GATCCAGCTGAGAGAGCCCA

P14: CAAACATCTCTGCACTTGTC

P17: TACCAAACGCAAGGAATTGA

P18: CCCAAGTGAAACTTGCTGCAG

P22: AGGAGGATGACATTAGTCAACC

**B:** RT-PCR on *Atzip4-1* and *Atzip4-2* alleles

## B-1 B-2

**1 2 3 H_2_O**

**L 1 2 3 H2O**

RT-PCR on cDNA isolated from flower buds from *Atzip4-1* (lane 1), *Atzip4-2* (lane 2) or wild-type (lane 3) plants. L : Fermentas 1 kb DNA ladder

B-1: For *AtZIP4* amplification, a nested PCR was performed, first with primers P5 and P13, second with primers P12 and P14. Expected amplification size for wild-type cDNA sample : 250 bp, for genomic amplification : 430 bp.

B-2: cDNA were calibrated according to the expression of the adenine phosphoribosyltransferase-encoding gene (*APT*, Moffat et al. 1994, Gene. 143: 211-216).

**C :** PCR characterisation of the *Atzip4-3* allele

**P3/P4**

**P3R/P5**

**P3R/P12**

**P5/P13**

**P6/P17**

**P6/P7**

**L**

**1 2 3 4**

**1 2 3 4**

**1 2 3 4**

**1 2 3 4**

**1 2 3 4**

**1 2 3 4**

**APT**

**4 3 2 1**

**L**

Genomic DNA amplification was performed using a series of primer combination covering the whole *AtZIP4* coding sequence (see Fig S1A) or using a set of primer amplifying the control *APT* gene. 1: DNA from a fertile heterozygous *AtZIP4-3^+/-^* plant*.* 2: DNA from a sterile mutant *Atzip4-3^-/-^* plant. 3: DNA from a wild-type plant. 4: water. Not shown: PCR with P18 and P22 showing also no amplification on mutant DNA.
