# Supplementary figures and images for: Zip4/Spo22 Is Required for Class I CO Formation but Not for Synapsis Completion in Arabidopsis thaliana
Source: PLoS Genet. 2007 May 25;3(5):e83. doi: 10.1371/journal.pgen.0030083 (PMC1877879; doi:10.1371/journal.pgen.0030083)

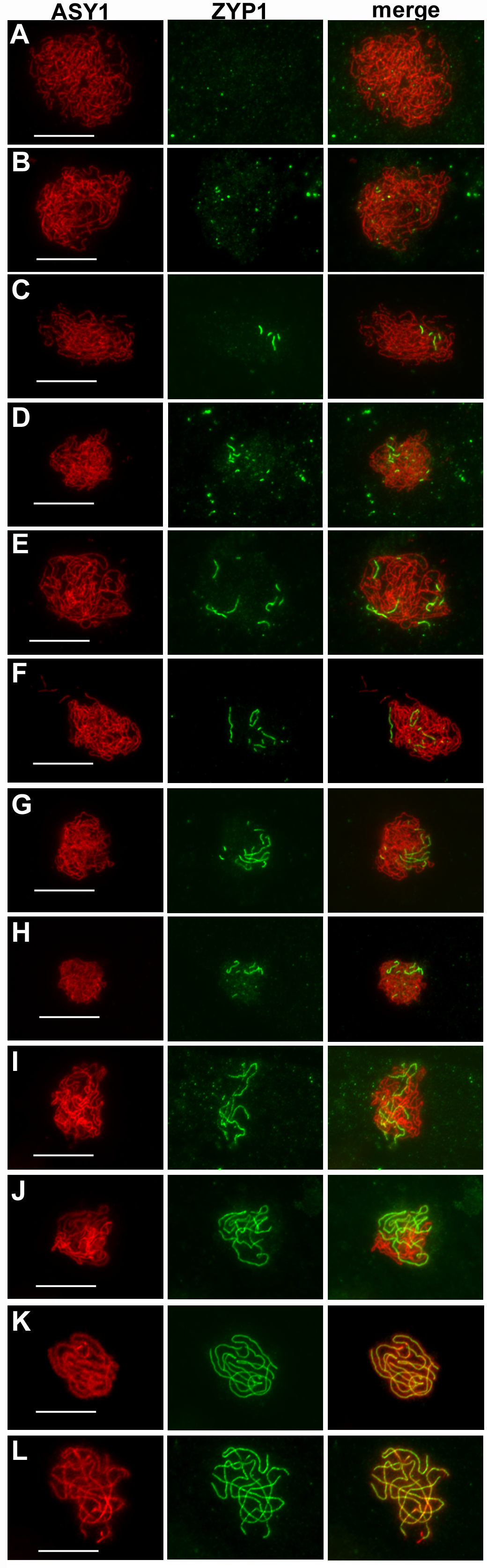

Supplement: Figure S2 — Coimmunolocalization of ASY1 (red) and ZYP1 (green) in wild-type PMCs. Prophase I cells showing increasing level of synapsis (according to anti-ZYP1 labeling) are shown: absence of synapsis (leptotene, A), partial synapsis (zygotene, B–J), and full synapsis (pachytene, K–L). For each cell, single labeling is shown as well as the merge signal. Bar, 10 μm. (2.2 MB TIF) [file pgen.0030083.sg002.tif]

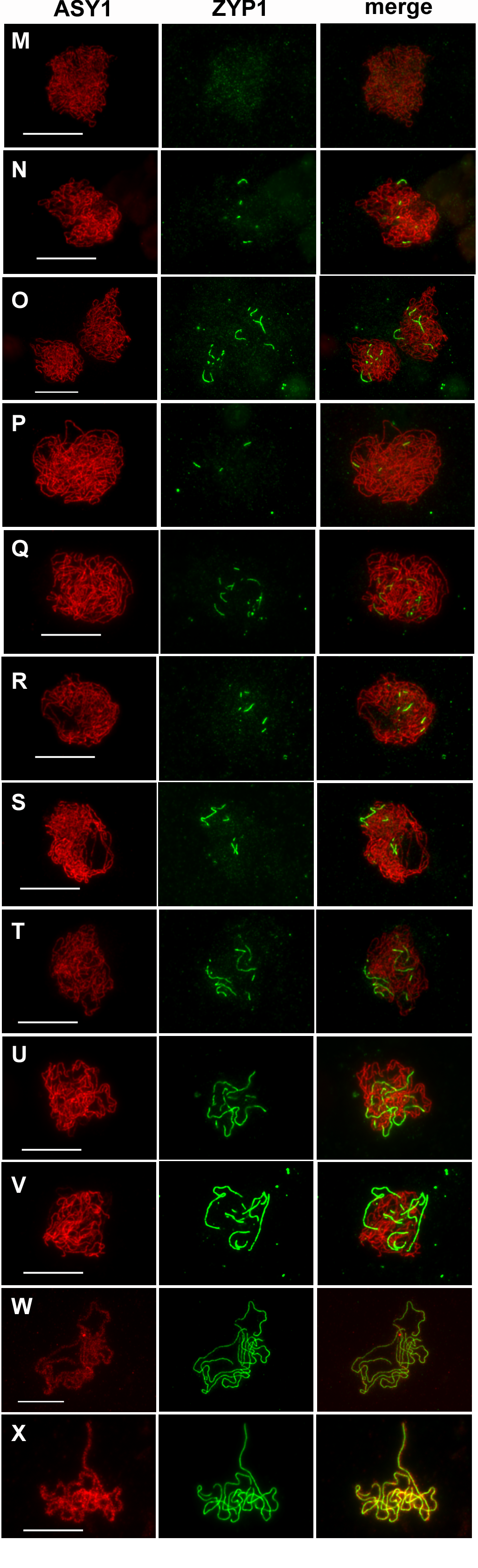

Supplement: Figure S3 — Coimmunolocalization of ASY1 (red) and ZYP1 (green) in Atzip4-1 PMCs. Prophase I cells showing increasing level of synapsis (according to anti-ZYP1 labeling) are shown: absence of synapsis (leptotene, M), partial synapsis (zygotene, N–V), and full synapsis (pachytene, W–X). For each cell, single labeling is shown as well as the merge signal. Bar, 10 μm. (2.2 MB TIF) [file pgen.0030083.sg003.tif]
